# Profile of Endothelin-1, Nitric Oxide, and Prostacyclin Levels in Pulmonary Arterial Hypertension Related to Uncorrected Atrial Septal Defect: Results from a Single Center Study in Indonesia

**DOI:** 10.1155/2020/7526508

**Published:** 2020-01-07

**Authors:** Lucia Kris Dinarti, Anggoro Budi Hartopo, Dyah Wulan Anggrahini, Ahmad Hamim Sadewa, Budi Yuli Setianto, Abdus Samik Wahab

**Affiliations:** ^1^Department of Cardiology and Vascular Medicine, Faculty of Medicine, Public Health and Nursing, Universitas Gadjah Mada–Dr. Sardjito Hospital, Yogyakarta, Indonesia; ^2^Department of Biochemistry, Faculty of Medicine, Public Health and Nursing, Universitas Gadjah Mada, Yogyakarta, Indonesia; ^3^Department of Child Health, Faculty of Medicine, Public Health and Nursing, Universitas Gadjah Mada–Dr. Sardjito Hospital, Yogyakarta, Indonesia

## Abstract

**Methods:**

The study design was cross-sectional. The subjects were adult uncorrected secundum ASD with PAH. Pulmonary artery pressure was measured with right heart catheterization. Pulmonary venous blood was obtained during catheterization for measuring endothelin-1, prostacyclin, and nitric oxide. Correlation tests were performed to determine any association between biomarkers and mean pulmonary artery pressure (mPAP). The levels of biomarkers were compared based on the severity of PAH. Statistical significance was determined at *p* < 0.05.

**Results:**

Forty-four subjects were enrolled in this study. Endothelin-1 level and mPAP had significant moderate positive correlation (*r* = 0.423 and *p* value = 0.004). However, no significant correlation was observed between prostacyclin, nitric oxide levels, and mPAP. The pattern of endothelin-1, prostacyclin, and nitric oxide was distinctive. Levels of endothelin-1 were incrementally increased from mild, moderate, to severe PAH. The levels of prostacyclin and nitric oxide had similar pattern in association with the severity of PAH, which was increased in mild-to-moderate PAH but decreased in severe PAH.

**Conclusions:**

There was a distinctive pattern of endothelin-1, prostacyclin, and nitric oxide based on severity of PAH in adult uncorrected ASD. Significant correlations existed between endothelin-1 and the severity of PAH and mPAP.

## 1. Introduction

Pulmonary arterial hypertension (PAH) is an increase in mean pulmonary artery pressure (mPAP) exceeding 25 mmHg at rest with normal pulmonary artery wedge pressure (PAWP) and elevated pulmonary vascular resistance (PVR) by more than 3 Wood units, owing to the restriction in pulmonary vascular flow [1]. The pathomechanisms of PAH involve pulmonary vascular modification, i.e., intimal endothelial dysfunction, reduced apoptosis, and proliferation ratio of pulmonary artery smooth muscle cells in medial layers, and increased adventitial thickening [2]. These vascular changes produce vasoactive agents to facilitate pulmonary blood flow through pulmonary tissue. Increased production of vasoconstrictor agents, such as endothelin-1 and thromboxane, as well as a decreased production of vasodilator agents, such as prostacyclin and nitric oxide, are hallmarks of PAH [3]. Compared to idiopathic PAH, the dynamics of vasoactive biomarkers may have a different pattern in conditions with chronically elevated pulmonary flow, such as in congenital heart disease (CHD).

CHD with left-to-right shunt may cause PAH. The prevalence of PAH among CHD is varied and influenced by defect size and location [4]. Atrial septal defect (ASD) is a pretricuspid shunt which causes pulmonary blood overflow and leads to endothelial dysfunction and pulmonary vascular modification [5]. In uncorrected ASD, the functional disturbance precedes the anatomical defect of pulmonary vasculatures; therefore, PAH may develop later in adulthood [5]. Among CHD with left-to-right shunt and PAH, the proportion of ASD as the underlying defect is 30%, many of which is secundum ASD [6]. Unfortunately, secundum ASD is also the most commonly encountered CHD in adults, largely due to being undiagnosed in childhood.

Three main pathophysiological pathways have been recognized as the major components in the development of PAH, i.e., endothelin-1, nitric oxide, and prostacyclin pathways [7]. Interactions among these pathways influence the development and progression of PAH. However, the role and the dynamics of their vasoactive components in secundum ASD-associated PAH remain to be elucidated. The proposed mechanism of functional and anatomical disturbances of physiological pathways in PAH requires corroboration in the context of uncorrected secundum ASD-associated PAH. The study aimed to investigate the relation between vasoactive agents, such as endothelin-1, nitric oxide, and prostacyclin plasma levels, and severity of PAH in adult patients with uncorrected secundum ASD.

## 2. Methods

### 2.1. Subjects

We conducted a cross-sectional study using the COngenital HeARt Disease in adult and Pulmonary Hypertension Registry of Universitas Gadjah Mada, Dr. Sardjito Hospital, Yogyakarta, Indonesia (COHARD-PH Registry). The pilot study of this registry had been published elsewhere [8].

The inclusion criteria for the current study were as follows: (1) male and female patients aged ≥ 18 years old, (2) patients newly diagnosed with large secundum ASD (diameter of defect ≥ 20 mm), (3) patients diagnosed with PAH by right heart catheterization (RHC), and (4) patients gave informed consent to participate in the study. The exclusion criteria were as follows: (1) patients had underwent ASD closure (device or surgery), (2) patients have been treated with specific PAH medication, (3) patients with comorbidities, such as other CHDs, significant (moderate to severe) valvular heart disease (VHD), and chronic lung/respiratory disease, and (4) patients with creatinine level >2.0 mg/dL.

Patients gave informed consent and were enrolled consecutively. Transthoracal echocardiography (TTE) and transesophageal echocardiography (TOE) were conducted in the echo-lab of Dr. Sardjito Hospital to diagnose large secundum ASD, estimate probability of PH, and exclude other CHD(s) and VHD(s). Physical examination and chest X-ray excluded patients with chronic lung/respiratory disease, such as chronic obstructive pulmonary disease, intermittent/persistent asthma bronchial, bronchiectasis, and pulmonary tuberculosis. Pulse oximetry measured peripheral O_2_ saturation. Patients fulfilling research criteria were sent for right heart catheterization (RHC) procedure in the cath-lab of Dr. Sardjito Hospital to diagnose PAH and measure hemodynamic parameters. This study was approved by the Medical and Health Research Ethics Committee of the Faculty of Medicine Universitas Gadjah Mada, Dr. Sardjito Hospital.

### 2.2. Procedures

Experienced sonographer performed the TTE using Vivid 7GE (G.E. Healthcare, U.S.A). Two cardiology consultants validated the TTE results. Two-dimensional echocardiography was performed according to standard practice. Heart chamber dimension, right and left ventricular function, defect diameter and type, and flow direction were measured based on standard procedures [9]. Estimated right ventricle systolic pressure (RVSP) was calculated based on the summation of Doppler spectral tricuspid valvular regurgitant gradient (TVRG) and estimated right atrial pressure, which was calculated from inferior vena cava diameter and its collapsibility index [10].

Cardiology consultants examined the diameter, type, and size of the defect, anatomy of ASD, and concomitant CHDs or VHDs by TOE using Vivid 7GE (G.E. Healthcare, U.S.A). The cardiologist consultants had >80% agreement in interpreting the TTE and TOE results.

Patients underwent RHC in the cath-lab operated by cardiology consultant using Xper Cardio Physiomonitoring System 5 (Philips, The Netherlands). Following aseptic preparation and anesthetic application, multipurpose catheter was inserted into the right femoral vein to the right atrium via inferior vena cava and proceeded into the left atrium by crossing through the ASD and was placed in the pulmonary veins. The Swan-Ganz catheter was inserted into right femoral veins through the right atrium and into the right ventricle and was placed in the pulmonary arteries. Pressure and oxygen saturation (Avoximeter R 1000E, U.S.A) were measured in each of the cardiac chambers, great arteries, and veins in accordance with standard procedures. The mPAP and PAWP were measured with Swan-Ganz catheter. PAH was defined as mPAP value ≥ 25 mmHg, PAWP value < 15 mmHg, and PVR >3 Wood units. Cardiac output measurement was calculated using indirect Fick method based on estimated oxygen consumption per square body surface area [11]. The PVR and flow ratio were calculated with the standard formula [11]. Severity of PAH was arbitrarily grouped based on mPAP, i.e., mPAP 25–40 mmHg as mild PAH, mPAP 41–60 mmHg as moderate PAH, and mPAP >60 mmHg as severe PAH.

### 2.3. Blood Examination

Prior to RHC, the peripheral venous blood sample was collected to measure haemoglobin, hematocrit, and creatinine levels. The measurements were performed in the hospital central laboratory using the standard protocol. During RHC, blood samples from pulmonary veins were collected into EDTA-containing polypropylene tubes. The sample was left in room temperature for 30 min and centrifuged at 4000 r.p.m for 10 min at 4°C. The supernatant was separated, stored, and pooled at −80°C until measurement of endothelin-1, prostacyclin, and nitric oxide. Endothelin-1 concentration was measured by ELISA and was read on the plate reader at 450 nm (Enzo, Japan). Because prostacyclin has a very short half-life, 6-keto-prostaglandin-F1*α* was measured as prostacyclin metabolites by EIA (Enzo, Japan). Nitric oxide concentration was measured based on Griess reaction based on colorimetrics (Enzo, Japan). All measurements were performed according to manufacturer's instructions.

### 2.4. Statistical Analysis

We described continuous data in means and standard deviation or median and interquartile range based on data distribution (data distribution normality was assessed with Kolmogorov–Smirnov test). We described categorical data in percentage. The correlation between continuous data was analyzed with Pearson correlation test (for normally distributed data) or Spearman correlation test (for nonnormally distributed data). We analyzed comparisons of continuous and categorical data among the groups with ANOVA and chi-square tests, respectively. The *p* value<0.05 was considered statistically significant. The statistical analysis was performed by using IBM SPSS 21 software package.

## 3. Results

### 3.1. Subject Characteristics

We conducted this study from January to November 2015 and enrolled forty-four subjects who met study criteria. The subjects had mean age of 39.5 ± 13.3 years old and largely comprised female (90.9%). The median peripheral O_2_ saturation was 96%. Based on WHO functional class classification, the subjects were predominantly in WHO functional class II (79.5%), while only a minority (4.5%) was in functional class III. The mean haemoglobin level was 13.4 g/dL, and mean hematocrit concentration was 40%. The mean endothelin-1 level was 674.29 ± 405.28 pg/dL. The median prostacyclin level was 413.5 pg/dL. The mean nitric oxide level was 325.49 ± 77.56 *μ*mol/L.  Table 1 shows the clinical and laboratory characteristics of the subjects.

TTE and TOE showed that the mean minimum ASD diameter was 2.5 ± 0.6 cm, and maximum ASD diameter was 2.7 ± 0.9 cm. We detected enlargement of both right atrium (mean diameter 4.8 ± 0.7 cm) and right ventricle (mean diameter 4.7 ± 0.5 cm). Left heart dimensions were within normal limit. The majority of subjects has normal right ventricle function as indicated by mean TAPSE value of 2.5 ± 0.5 cm. An increase in pulmonary artery pressure was predicted with increased tricuspid valve regurgitant gradient (TVRG) (mean pressure 78.0 ± 28.5 mmHg) and right ventricular systolic pressure (mean pressure 85.4 ± 28.2 mmHg). The majority of subjects (61.4%) have left-to-right shunt across the defect, whereas 38.6% subjects have already developed bidirectional shunt with predominant left-to-right shunt. Secondary tricuspid valve regurgitation was encountered in all subjects with various severities, with predominance of moderate regurgitation (54.5%).  Table 1 shows the echocardiography parameters of the subjects.

The hemodynamic data from RHC showed that the mean mPAP was elevated (51.0 mmHg). The mean PAWP was normal, i.e., 8.0 ± 4.4 mmHg. The median of PVR was 12.7 Wood units, which was classified as high resistance. The median value of pulmonary-to-systemic flow ratio was 1.7. Based on mPAP cutoff point, 13 subjects (29.5 %) had mild PAH, 22 subjects (50%) had moderate PAH, and 9 subjects (20.5 %) had severe PAH.  Table 1 shows the hemodynamic parameters recorded during RHC.

### 3.2. Correlation between Biomarkers and mPAP

**Figure 1:**
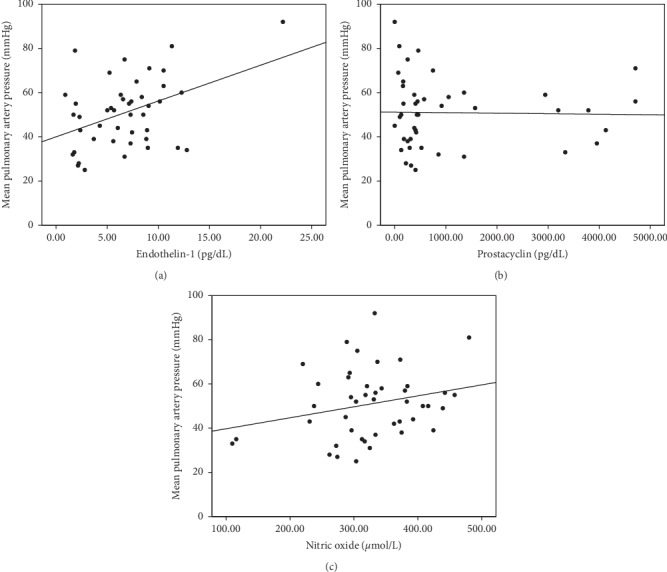
(a–c). Scatter plots indicate the significantly moderate positive correlation between endothelin-1 level and mPAP (*r* = 0.423 and *p* value = 0.004) (Figure 1(a)). No significant correlation is observed between prostacyclin and mPAP (*r* = −0.058 and *p* value = 0.710) (Figure 1(b)) and nitric oxide and mPAP (*r* = 0.248 and *p* value = 0.105) (Figure 1(c)).

Endothelin-1 level and mPAP had significant moderate positive correlation, with correlation coefficient (Pearson) *r* = 0.423 and *p* value = 0.004. In contrast, we observed no significant correlation between prostacyclin level and mPAP (Spearman, *r* = −0.058 and *p* value = 0.710) nor between nitric oxide levels and mPAP (Pearson, *r* = 0.248 and *p* value = 0.105). The scatter plots indicating these correlations are depicted in Figure 1.

### 3.3. Pattern of Biomarkers Based on PAH Severity

Based on mPAP value, the PAH severity was divided into three categories. The classification of PAH severity is depicted in Table 2. There was a statistically significant association between ages and PAH severities, with younger subjects associated with more severe PAH. Systolic blood pressure was significantly reduced in severe PAH, and diastolic blood pressure also tended to be lower. Peripheral O_2_ saturation was significantly decreased in moderate and severe PAH compared with mild PAH. Haemoglobin and hematocrit were significantly elevated in severe PAH. The TTE calculation of TVRG and RVSP was heightened in the more severe PAH. Left atrial and ventricle dimensions were reduced in the more severe PAH. From RHC parameters, PVR was increased in the more severe PAH, whereas flow ratio was significantly depressed.

**Figure 2:**
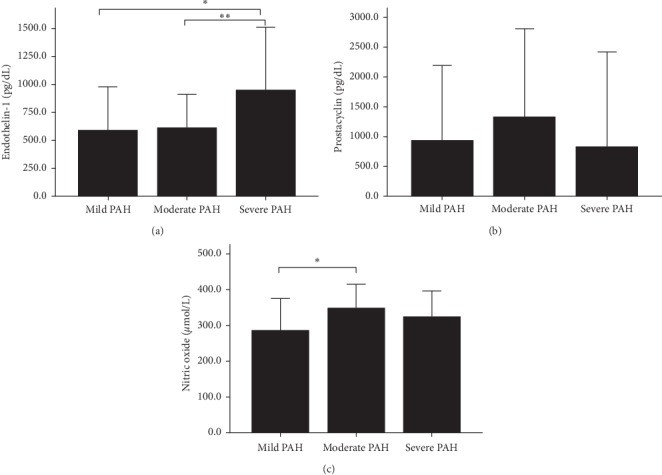
(a–c). Bar graphs indicate the distinctive pattern of biomarkers, i.e., endothelin-1, prostacyclin, and nitric oxide, according to the severity of PAH. Endothelin-1 is constantly increased from mild, moderate to severe PAH (Figure 2(a)). Post hoc analysis shows significant increase in endothelin-1 level in severe PAH as compared with mild (*p*=0.039) and moderate PAH (*p*=0.036). There was an increased level of prostacyclin in mild-to-moderate PAH and decreased level in severe PAH (Kruskal–Wallis, *p* value = 0.128) (Figure 2(b)). Similar pattern was observed in the level of nitric oxide which was increased in mild-to-moderate PAH, whereas decreased in severe PAH (one-way ANOVA, *p* value = 0.064) (Figure 2(c)). Post hoc analysis showed significant increase from mild to moderate PAH (*p*=0.020).

Further analysis on the pattern of biomarkers according to the severity of PAH revealed that the pattern of endothelin-1, prostacyclin, and nitric oxide was distinctive. The level of endothelin-1 constantly rose from subjects with mild PAH, moderate PAH, to severe PAH (mean ± SD: 587.52 ± 388.93 pg/dL, 613.57 ± 297.38 pg/dL to 948.09 ± 563.13 pg/dL, consecutively, *p* value = 0.071). The level of prostacyclin and nitric oxide had a similar pattern in association with severity of PAH. There was higher level of prostacyclin in subjects with mild-to-moderate PAH (median: 319.78 pg/dL to 453.76 pg/dL, respectively) and decreased level in subjects with severe PAH (median: 166.10 pg/dL) (Kruskal–Wallis test, *p* value = 0.128). Similar pattern was observed in the level of nitric oxide which was elevated in subjects with mild-to-moderate PAH (mean ± SD: 286.18 ± 88.42 *μ*mol/L to 349.12 ± 65.86 *μ*mol/L, respectively), whereas it was lowered in subjects with severe PAH (mean ± SD: 324.51 ± 71.92 *μ*mol/L) (all differences *p* value = 0.064). Table 2 and Figure 2 show the distinctive pattern of endothelin-1, nitric oxide, and prostacyclin based on PAH severity.

### 3.4. Correlation between Other Parameters and mPAP

Table 3 shows the characteristics/parameters which associate with mPAP. Parameters with significant inverse/negative correlation with mPAP were age, systolic and diastolic blood pressure, peripheral O_2_ saturation, left atrial dimension, LVIDd, and flow ratio. On the contrary, haemoglobin, TVRG, and RVSP had significant positive correlation with mPAP. Among these parameters, age and flow ratio had higher correlation coefficient, indicating stronger inverse/negative correlation with mPAP.

## 4. Discussion

To our knowledge, this is the first study to examine the profile of biomarkers in PAH related to uncorrected ASD. Our study revealed that among vasoactive agents, endothelin-1 has significant positive and moderate correlation with mPAP. Endothelin-1 level rises incrementally with PAH severity. Meanwhile, vasodilator substances, such as prostacyclin and nitric oxide, exhibit different fluctuation patterns. It increases in mild-to-moderate PAH, but decreases in severe PAH. It indicates that in PAH due to CHD with the left-to-right shunt, rising flow in the pulmonary vasculature will stimulate physiologic responses such as higher levels of the vasoconstrictor endothelin-1 and vasodilators such as nitric oxide and prostacyclin as a counterbalance. However, this physiologic response is blunted in severe PAH, indicated by discrepancy between vasoconstrictor and vasodilator interaction, possibly due to advanced pulmonary vascular remodeling.

We found a significant positive and moderate correlation between endothelin-1 level and mPAP. There was also an incremental increasing of endothelin-1 level in accordance with the severity of PAH, based on mPAP graded value. Li et al. [12] reported a similar result after investigating 30 healthy subjects and 58 PAH patients. Recent study comparing endothelin-1 level from 80 adults ASD and 19 healthy subjects found that endothelin-1 level was significantly higher in unclosed ASD than in that with closed defects [13].

In this study, prostacyclin level was not significantly correlated with mPAP. Prostacyclin level was higher in mild and moderate PAH before declining in severe PAH. In an experimental study, Li et al. [14] investigated the effect of blood flow on the mediator release including prostacyclin and showed similar pattern as in our human study. In contrast to physiological shear stress, pathological shear stress (both low and high flow) diminished release of prostacyclin in circulation [14]. This finding revealed that physiological flow shear stress stimulates vasodilator release, whereas low or high pathological flow shear stress lessens vasodilator release. The balance between vasoconstrictors and vasodilators is disrupted by pathological flow. Moreover, Tuder et al. [15] proved that CHD results in more fluctuating production of prostacyclin synthase in three different diameters of pulmonary arteries. This study demonstrated that severe PAH patients expressed significantly lower levels of prostacyclin enzyme. Therefore, the reduced or absence of prostacyclin production might be the marker for severe PAH [15]. We suggest that advanced pulmonary vascular remodeling contributes to reduced prostacyclin synthase enzymes and subsequently lower prostacyclin level.

We found that nitric oxide levels did not significantly correlate with mPAP. Nitric oxide has a propensity to be higher in mild-to-moderate PAH. Strikingly, it dropped in severe PAH. Similar pattern was seen in that of prostacyclin. Consistently, Kiettisanpipop et al. [16] reported differences in nitric oxide levels between severe and nonsevere PAH (cutoff point 60 mmHg). Nitric oxide level was strongly correlated with mild and moderate PAH but was lowered in subjects with severe PAH [16].

In an experimental study using the aortocaval shunt model to mimic left-to-right shunt defect and PAH, there was a prominent rise of eNOS production in the animal model with aortocaval shunt compared with the control [17]. It indicates that eNOS, as well as nitric oxide, was produced as a consequence of continuous overflow to the pulmonary artery [17]. Another study with a similar model and observation of 12 weeks showed a significant increase of eNOS compared with sham controls [18]. The result proved that eNOS greatly participates in remodeling process of pulmonary vascular before PAH development. An *in vitro* study reported that acute changes in flow or shear stress contribute to the activation of eNOS and nitric oxide [14]. Tworetzky et al. [19] investigated the acute effect of pulmonary flow alteration in humans. Nitric oxide levels were measured in ASD patients before and after closure by device [19]. It confirmed that nitric oxide levels decreased after defect closure. It was demonstrated that nitric oxide levels were significantly higher in children with congenital heart disease and PAH compared with non-PAH children [20]. Nevertheless, nitric oxide level based on the severity of PAH was not investigated.

Our study intended to elucidate the role of three biomarkers, namely, endothelin-1, prostacyclin, and nitric oxide, in the occurrence of PAH in ASD. Prostacyclin and nitric oxide levels were higher in mild and moderate PAH due to physiological response of pulmonary vasculatures as a consequence of continuous shear stress, though their levels decline as mPAP rises above 60 mmHg. On the contrary, vasoconstrictor endothelin-1 steadily and incrementally increased along with rising pulmonary artery pressure.

Among biomarkers, there are interactions in CHD and PAH. Shear stress due to overflow inevitably triggers nitric oxide production as well as endothelin-1 inhibition from endothelium. Between endothelin-1 and nitric oxide, an interaction is linked through several mechanisms such as cGMP and ET_B_ receptor activation [21]. The sustained interaction between endothelin-1 and ET_B_ receptors will generate deleterious effects on cell function even though it may initially produce nitric oxide in the early stage of PAH [21]. Endothelin-1 stimulates prostanoid formation, including prostacyclin, through the cyclooxygenase-dependent mechanism in endothelial cells via the ET_A_ receptor [22]. Prostacyclin triggers pulmonary artery relaxation by stimulating nitric oxide release and synergistically interacts with nitric oxide in smooth muscle cells. However, we did not find significant correlation among biomarkers in mild, moderate, or severe PAH, indicating more complex mechanisms in the pathobiology of CHD and PAH.

Interestingly, we found that female patients were predominant, and younger age was associated with more severe PAH. In this study, female subjects accounted for 90.9% of all ASD subjects, in line with previous studies showing that ASD was more prevalent in women. The CONCOR registry reported 62% of ASD patients were females [23]. Humenberger et al. [24] stated that women comprise 69.5% of all ASD patients. This female predominance is also recognized by Euro Heart Survey from 1998 to 2004 (68%) [25]. In our database, i.e., COHARD-PH Registry, among 350 adult ASD patients, 86% have been diagnosed PAH and 86% of those are women (unpublished data). Badesch et al. [26] from REVEAL Registry and Mair et al. [27] independently reported that 79.5% of PAH patients were females and were four times more susceptible to PAH than male patients. The inverse correlation between age and mPAP may indicate other factors contribute to the development of PAH in ASD patients. The presence of concomitant genetic background may predispose some patients to the development of PAH [28]. The pulmonary overflow due to the chronic left-to-right shunt is the triggering factor for development of PAH.

Given the distinctive patterns of three key biomarkers of PAH in our study, we speculate that in the early phase of PAH (mild-to-moderate PAH), the physiologic response of endothelin-1 still exists, such that the therapy with endothelin-1 receptor antagonists is important to reduce pulmonary artery pressure in early PAH. In the case of uncorrected ASD, the reduced pulmonary artery resistance, i.e., decreased mPAP and PVRI, may lead to the possibility of defect closure. On the contrary, prostacyclin analogue and PDE-5 inhibitor have their most impact in the later phase of PAH, as endogenous prostacyclin level is diminished.

## 5. Limitation of the Study

The statistical power of this study can be increased by adding more subjects. Causality between the biomarkers profile and pulmonary hypertension in unrepaired ASD is still unknown as this is a cross-sectional study. Further investigation with prospective cohort design could produce a more representative pattern of each biomarker and its relation with PAH severity.

## 6. Conclusion

In conclusion, there is a distinctive pattern among endothelin-1, nitric oxide, and prostacyclin in correlation with the severity of PAH in adult uncorrected ASD. A significantly positive correlation exists between endothelin-1 and the severity of PAH and mPAP. Meanwhile, nitric oxide and prostacyclin are increased in mild-to-moderate PAH then decreased in severe PAH. The flow ratio and age are inversely correlated with the severity of PAH.

## Figures and Tables

**Table 1:** Clinical, laboratory, echocardiography, and hemodynamics characteristics of all subjects.

The characteristics	All subjects *n* = 44
*Clinical and laboratory data*	
Age (years), mean ± SD	39.5 ± 13.3
Sex, *n* (%)	
Male (%)	4 (9.1)
Female (%)	40 (90.9)
SBP (mmHg), median (IQR)	101 (95–120)
DBP (mmHg), median (IQR)	70 (70–80)
Peripheral O_2_ saturation (%), median (IQR)	96 (93–98)
W.H.O functional class, *n* (%)	
I	7 (15.9)
II	35 (79.5)
III	2 (4.5)
Haemoglobin (g/dL), mean ± SD	13.4 ± 1.6
Hematocrit (%), mean ± SD	40.0 ± 4.8
Creatinine (g/dL), mean ± SD	0.8 ± 0.2
Endothelin-1 (pg/dL), mean ± SD	674.29 ± 405.28
Prostacyclin (pg/dL), median (IQR)	413.51 (190.13–1280.89)
Nitric oxide (*µ*mol/L), mean ± SD	325.49 ± 77.56

*Echocardiography data*	
Minimum ASD diameter (cm), mean ± SD	2.5 ± 0.6
Maximum ASD diameter (cm), mean ± SD	2.7 ± 0.9
Right atrial dimension (cm), mean ± SD	4.8 ± 0.7
Right ventricle dimension (cm), mean ± SD	4.7 ± 0.5
Left ventricle ejection fraction (%),mean ± SD	71.2 ± 10.1
TAPSE (cm), mean ± SD	2.5 ± 0.5
TVRG (mmHg), mean ± SD	78.0 ± 28.5
RVSP (mmHg), mean ± SD	85.4 ± 28.2
Left atrial dimension (cm), median (IQR)	3.3 (2.9–3.7)
LVIDd (cm), median (IQR)	3.5 (3.1–3.8)
Tricuspid valve regurgitation, *n* (%)	
Mild	3 (6.8)
Moderate	24 (54.5)
Severe	17 (38.6)
Shunt direction, *n* (%)	
Left to right	27 (61.4)
Bidirectional	17 (38.6)

*Hemodynamics RHC data*	
Mean pressure (mmHg)	
Aorta, mean ± SD	103.6 ± 15.0
RV systolic, median (IQR)	86.0 (64.0–103.0)
Pulmonary artery, mean ± SD	51.0 ± 15.2
Pulmonary artery wedge pressure (mmHg)	8.0 ± 4.4
O_2_ saturation (%)	
Aorta, median (IQR)	91.0 (85.0–94.0)
Mixed vein, median (IQR)	60.6 (55.0–64.0)
Left atrium, median (IQR)	90.5 (85.0–93.0)
Right atrium, mean ± SD	73.0 ± 9.4
Pulmonary vein, median (IQR)	95.5 (93.0–96.5)
Pulmonary artery, mean ± SD	77.0 ± 10.0
PVR (Woods unit), median (IQR)	12.7 (3.9–21.3)
Flow ratio, median (IQR)	1.7 (1.3–2.8)
PAH severity, *n* (%)	10 (22.7)
Mild	13 (29.5%)
Moderate	22 (50.0%)
Severe	9 (20.5%)

IQR: interquartile range, SBP: systolic blood pressure, DBP: diastolic blood pressure, ASD: atrial septal defect, TAPSE: tricuspid annular plane systolic excursion, TVRG: tricuspid valve regurgitant gradient, RVSP: right ventricular systolic pressure, LVIDd: left ventricle internal diastolic diameter, RHC: right heart catheterization, PVR: pulmonary vascular resistance, mPAP: mean pulmonary artery pressure, PAH: pulmonary artery hypertension, and RV: right ventricle.

**Table 2:** Clinical, echocardiography, and hemodynamics characteristics of the subjects based on PAH Severity.

The characteristics	Mild PAH *n* = 13	Moderate PAH *n* = 22	Severe PAH *n* = 9	*p* value^*∗*^
*Clinical and laboratory data*				
Age (years), mean ± SD	48.3 ± 10.9	38.2 ± 13.1	30.1 ± 9.4	0.030
Sex, *n* (%)				0.467
Male (%)	2 (15.4)	2 (9.1)	0 (0)	
Female (%)	11 (84.6)	20 (90.9)	9 (100)	
SBP (mmHg), median (IQR)	115.1 ± 14.2	105.1 ± 15.4	97.0 ± 7.4	0.014
DBP (mmHg), median (IQR)	74.1 ± 9.8	73.2 ± 13.2	68.3 ± 9.7	0.488
O_2_ saturation (%), median (IQR)	97.6 ± 1.7	93.5 ± 3.6	93.0 ± 4.4	0.002
W.H.O functional class, *n* (%)				0.005
I	5 (38.5)	2 (9.1)	0 (0)	
II	8 (61.5)	20 (90.9)	7 (77.8)	
III	0 (0)	0 (0)	2 (22.2)	
Haemoglobin (g/dL), mean ± SD	13.5 ± 1.3	13.4 ± 2.1	15.5 ± 2.4	0.029
Hematocrit (%), mean ± SD	40.8 ± 3.6	40.4 ± 6.7	46.3 ± 7.5	0.053
Creatinine (g/dL), mean ± SD	0.8 ± 0.3	0.8 ± 0.2	0.8 ± 0.1	0.917
Endothelin-1 (pg/dL), mean ± SD	587.52 ± 388.93	613.57 ± 297.38	948.09 ± 563.13	0.071
Prostacyclin (pg/dL), median (IQR)	319.78 (236.20–319.82)	453.76 (383.01-1572-44)	166.10 (85.14-900-31)	0.128
Nitric oxide (*µ*mol/L), mean ± SD	286.18 ± 88.42	349.12 ± 65.86	324.51 ± 71.92	0.064

*Echocardiography data*				
Minimum ASD diameter (cm), mean ± SD	2.4 ± 0.5	2.5 ± 0.6	2.6 ± 0.7	0.761
Maximum ASD diameter (cm), mean ± SD	2.5 ± 0.9	2.6 ± 1.0	2.8 ± 0.7	0.620
Right atrial dimension (cm), mean ± SD	4.8 ± 0.5	4.9 ± 0.8	4.3 ± 0.4	0.065
Right ventricle dimension (cm), mean ± SD	4.5 ± 0.3	4.8 ± 0.6	4.8 ± 0.5	0.263
Ejection fraction (%), mean ± SD	68 ± 11	71 ± 9	74 ± 11	0.398
TAPSE (cm), mean ± SD	2.6 ± 0.6	2.4 ± 0.5	2.3 ± 0.5	0.375
TVRG (mmHg), mean ± SD	50.5 ± 16.7	82.6 ± 19.9	106.2 ± 27.1	<0.001
RVSP (mmHg), mean ± SD	58.0 ± 16.4	90.1 ± 19.2	113.3 ± 27.1	<0.001
Left atrial dimension (cm), mean ± SD	3.9 ± 0.8	3.3 ± 0.6	3.0 ± 0.6	0.009
LVIDd (mm), mean ± SD	3.9 ± 0.8	3.5 ± 0.5	3.2 ± 0.4	0.020
Tricuspid valve regurgitation, *n* (%)				0.065
Mild	2 (15.4)	1 (4.5)	0 (0)	
Moderate	10 (76.9)	9 (40.9)	5 (55.6)	
Severe	1 (7.7)	12 (54.5)	4 (44.4)	
Shunt direction, *n* (%)				0.021
Left to right	12 (92.3)	10 (45.5)	5 (55.6)	
Bidirectional	1 (7.7)	12 (54.5)	4 (44.4)	

*Hemodynamics RHC data*				
Mean pressure (mmHg)				
Aorta, mean ± SD	114.4 ± 22.5	100.9 ± 14.9	95.1 ± 12.3	0.028
RV systolic, mean ± SD	56.8 ± 11.3	89.9 ± 14.4	111.8 ± 22.8	<0.001
Pulmonary artery, mean ± SD	33.6 ± 4.6	51.6 ± 6.0	73.9 ± 9.0	<0.001
Pulmonary artery wedge pressure (mmHg)	11.6 ± 6.5	7.7 ± 4.7	10.8 ± 5.4	0.094
O_2_ saturation (%)				
Aorta, mean ± SD	93.3 ± 1.5	87.5 ± 6.1	85.2 ± 7.4	0.004
Mixed vein, mean ± SD	61.2 ± 19.3	55.5 ± 13.5	58.4 ± 4.2	0.530
Left atrium, mean ± SD	85.7 ± 25.9	83.6 ± 19.4	89.1 ± 5.6	0.784
Right atrium, mean ± SD	81.6 ± 5.9	71.2 ± 9.4	68.3 ± 7.1	0.001
Pulmonary vein, mean ± SD	95.5 ± 1.3	93.2 ± 6.1	94.1 ± 4.2	0.461
Pulmonary artery, mean ± SD	86.7 ± 4.9	74.8 ± 8.5	71.3 ± 7.9	<0.001
PVR (Woods unit), mean ± SD	3.2 ± 1.6	15.6 ± 3.2	26.8 ± 12.4	<0.001
Flow ratio, mean ± SD	3.4 ± 1.4	1.8 ± 0.8	1.5 ± 0.9	<0.001

^*∗*^One-way ANOVA or Kruskal–Wallis test for continuous variables or chi-squared test for categorical variable, comparison among PAH severity. QR: interquartile range, SBP: systolic blood pressure, DBP: diastolic blood pressure, ASD: atrial septal defect, TAPSE: tricuspid annular plane systolic excursion, TVRG: tricuspid valve regurgitant gradient, RVSP: right ventricular systolic pressure, LVIDd: left ventricle internal diastolic diameter, PVR: pulmonary vascular resistance, and mPAP: mean pulmonary artery pressure.

**Table 3:** The correlation between mPAP with other parameters.

Parameters	*r* value^*∗*^	*p* value^*∗*^
Age	−0.497	0.001
Systolic blood pressure	−0.462	0.002
Diastolic blood pressure	−0.316	0.037
Peripheral O_2_ saturation	−0.460	0.002
Haemoglobin	0.300	0.048
Hematocrit	0.281	0.065
Creatinine	0.000	0.997
Minimum ASD diameter	0.103	0.513
Maximum ASD diameter	0.105	0.496
Right atrial dimension	−0.263	0.092
Right ventricle dimension	0.112	0.479
Left ventricle ejection fraction	0.249	0.104
TAPSE	−0.277	0.069
TVRG	0.673	<0.001
RVSP	0.676	<0.001
Left atrial dimension	−0.330	0.029
LVIDd	−0.375	0.012
Flow ratio	−0.508	<0.001

^*∗*^Pearson correlation or Spearman correlation if applicable. ASD: atrial septal defect, TAPSE: tricuspid annular plane systolic excursion, TVRG: tricuspid valve regurgitant gradient, RVSP: right ventricular systolic pressure, and LVIDd: left ventricle internal diastolic diameter.

## Data Availability

The raw data and data set used to support the findings of this study have not been made available because of the confidentiality policy in our department. However, authors are open to any discussions regarding this research.

## Additional Points

All authors assume responsibility for all aspects of the reliability and freedom from bias of the data presented and their discussed interpretation.
